# Patterns of Alcohol Consumption in the PURE Poland Cohort Study and Their Relationship with Health Problems

**DOI:** 10.3390/ijerph18084185

**Published:** 2021-04-15

**Authors:** Katarzyna Zatońska, Piotr Psikus, Alicja Basiak-Rasała, Zuzanna Stępnicka, Maria Wołyniec, Andrzej Wojtyła, Andrzej Szuba, Katarzyna Połtyn-Zaradna

**Affiliations:** 1 Department of Social Medicine, Wroclaw Medical University, 50-345 Wrocław, Poland; katarzyna.zatonska@umed.wroc.pl (K.Z.); zuzanna.stepnicka@gmail.com (Z.S.); maria.wolyniec@umed.wroc.pl (M.W.); Katarzyna.poltyn-zaradna@umed.wroc.pl (K.P.-Z.); 2 Calisia University, 62-800 Kalisz, Poland; burmistrz@um.kepno.pl (P.P.); a.wojtyla55@gmail.com (A.W.); 3 Department of Angiology, Hypertension and Diabetology, Wroclaw Medical University, 50-556 Wroclaw, Poland; andrzej.szuba@umed.wroc.pl

**Keywords:** alcohol, cohort study, PURE

## Abstract

(1) Background: Alcohol is a leading risk factor of premature morbidity and mortality. The objective of this study was to investigate the patterns of alcohol consumption in the PURE Poland cohort study baseline. (2) Methods: A Polish cohort was enrolled in the baseline study in 2007–2010. The study group consisted of 2021 adult participants of urban and rural areas from the Lower Silesia voivodeship in Poland (747 men and 1274 women). (3) Results: In the overall study population, 67.3% were current drinkers, 10.3% were former drinkers, and 22.4% were abstainers. Current use of alcohol products was more prevalent in men (77.2%), people living in urban areas (73.0%), and people with a higher level of education (78.0%). The percentage of current drinkers decreased with increasing age (from 73.4% in 30- to 44-year-olds to 48.8% in participants aged 64 and more). The majority of participants (89.2%) declared a low level of alcohol intake. The chance of high level of intake of alcohol was four times higher in men than in women (OR 4.17; CI 1.64–10.6). The majority of participants (54.6%) declared most frequent consumption of low-alcohol drinks (beer, wine) and 21% declared most frequent consumption of spirits. Current drinkers had almost 1.5-fold higher odds of diabetes and cardiovascular diseases (CVD) than never drinkers (OR 1.49, CI 1.03–2.17; OR 1.66, CI 1.27–2.18, respectively). Former drinkers had higher odds for hypertension and CVD than never drinkers (1.73, CI 1.05–2.85; OR 1.76, CI 1.22–2.53, respectively). (4) Conclusions: In our cohort study, we observed several socio-demographic factors differentiating the patterns of alcohol consumption. The preventive programs should focus predominantly on men, people aged <45 years, and those with a higher level of education.

## 1. Introduction

Alcohol is included in many social, cultural, and religious practices. Widespread use of alcoholic beverages can have deleterious effects on health. Alcohol is a leading risk factor of premature morbidity and mortality. According to the World Health Organization (WHO), there were 2.3 billion current drinkers in 2016 worldwide [1]. It is estimated that 5.3% of overall global mortality can be attributed to excessive consumption of alcohol [1]. Based on to-date studies, there is a linear association between the level of alcohol consumption and all-cause mortality, especially cancer-related [2]. Aforementioned findings contradict previous observations, which suggested the protective impact of consumption of low to moderate amounts of alcohol [3,4]. The previous observations can be partially explained by an inadequate control for confounders and an association between light drinking and a healthier overall lifestyle [2]. Harmful use of alcohol increases the risk of premature mortality, as well as the risk of injuries, disability, and mental and psychological disorders. It is estimated that 5.5% of cancer cases worldwide can be attributed to the consumption of alcohol [5]. Moderate to high alcohol intake has been previously associated with cancers of the larynx, esophagus, pharynx, oral cavity, colorectum, and breast, but even light alcohol intake has been linked to cancers of the pharynx, oral cavity, esophagus, and breast [6,7]. The eastern European region has one of the highest burdens of disease attributed to alcohol with the contribution of alcohol use disorders, cardiovascular diseases, and injuries [8]. According to the European Health Interview Survey (EHIS) conducted in 2014, alcohol consumption in Poland was the most frequent in 30–49-year-olds [9]. In 2014 in Poland, 63% of women and 83% of men declared at least occasional consumption of alcoholic beverages. One in three adult men and one in seven adult women reported that they consumed alcohol at least once a week [9]. One of the most comprehensive reports on alcohol epidemiology across the European Union was prepared within the framework of the Joint Action on Reducing Alcohol Related Harm (RARHA) project [10]. According to the report, 86.4% of respondents in Poland declared consumption of alcohol in the previous 12 months. 

It is observed that consumption of alcohol might be differentiated by many socio-demographic factors, including sex, place of residence, and level of education [11,12,13,14,15,16]. According to the Global Burden of Disease (GBD) study [2], the attributable health burden caused by the excessive consumption of alcohol was three times higher in men than in women. In Poland in 2016, the average annual consumption of pure alcohol per capita in men was almost 3-fold higher than in women (23.8 L vs. 8.3 L, respectively) [12]. Moreover, the prevalence of heavy episodic drinking was much higher in men than in women. Recently published studies indicated a causal association between education attainment and alcohol drinking patterns [13]. A lower level of education in Poland has been associated with increased mortality caused by alcoholic liver diseases [17].

In 2016 worldwide, the most common types of alcoholic beverages were spirits (44.8%), beer (34.2%), and wine (11.7%) [1]. In contrast, in Poland, the most popular type of alcohol was beer. Based on the EHIS report, beer contributed to 70% of consumption of alcoholic beverages, whereas spirits to 20% and wine to 10% of overall consumption [9]. According to reports by The State Agency for the Prevention of Alcohol-Related Problems (PARPA), the average annual consumption of spirits, wine, and beer in 2019 per capita added up to 3.7 L, 6.2 L, and 97.1 L, respectively [18]. The average annual consumption of pure alcohol per capita in Poland in 2019 was 9.78 L. There has been a steady increase of average alcohol consumption in Poland since the 1990s. In 1993, the average annual consumption of pure alcohol per capita was approximately 6.52 L [18]. Between 1990 and 2007, the average alcohol consumption in Poland was significantly lower in comparison to other European Union (EU) countries [12]. The trend shifted after 2007, when the average alcohol consumption in Poland began to steadily increase, reaching the EU average, and then exceeding it about 2013–2014, and it has remained higher ever since [12]. The increasing trend of alcohol consumption in Poland poses a challenge for health policy makers. It is speculated that the slower rate of health improvement and slower decrease in premature mortality since 2000s in Poland can be attributed, among other factors, to increasing alcohol consumption [19,20,21]. A significant increase in mortality caused by alcohol-related liver cirrhosis was observed in this period of time [22]. In Poland, 14% of disability-adjusted life years (DALY) in men can be attributed to consumption of alcohol, which is a high value relative to the scale of the European Union [23]. Concomitantly, alcoholic beverages are very affordable and accessible in Poland [24]. 

A Polish cohort participates in the global Prospective Urban and Rural Epidemiological (PURE) study. The primary aim of the global PURE study was a longitudinal observation of modifiable and non-modifiable risk factors of non-communicable diseases. The urban–rural setting of the study enabled a comparison of risk factors between regions and tracking health inequalities. The objective of the here-presented study was to perform a sociodemographic analysis of the patterns of alcohol consumption in the PURE Poland cohort study baseline. Additionally, we assessed the prevalence of diabetes, hypertension, cardiovascular diseases (CVD), and select liver diseases in groups of participants differentiated by the attitude toward alcohol consumption. Poland is one of the most interesting countries in which to investigate alcohol consumption patterns, due to rapid changes in attitudes toward alcohol over the years. Our study is one of the few ongoing cohort studies in Poland, conducted with a consistent protocol and analyzing the socio-demographic characteristics of presented data with special emphasis on the place of residence (urban–rural). The number of prospective studies, similar in size in Poland, is limited. 

## 2. Materials and Methods

The Polish cohort was enrolled in the baseline study between 2007 and 2010. The enrollment to the Polish cohort of the PURE study was announced in mass media (local newspapers, television, radio). The volunteers who applied had to meet the criteria of age and place of residence (urban–rural). The participants were chosen to achieve a broadly representative sample of the community [25]. The baseline cohort consisted of 2036 inhabitants aged 30–85 years of urban and rural areas from Lower Silesia voivodeship in Poland. Data are collected every three years. The full protocol of the PURE study was previously described [25,26]. Every visit in the study center included a questionnaire study (individual health, family, household, food frequency, and international physical activity questionnaires), a blood draw, blood pressure measurement, a spirometry, and anthropometric measurements [26]. 

The presented article includes an analysis of the baseline data of the PURE Poland cohort study. Out of 2036 baseline participants, 15 were excluded from analysis of alcohol consumption patterns due to a lack of full data regarding alcohol consumption. A total of 2021 eligible participants (747 men and 1274 women) were included for those analyses. The analysis of the occurrence of diabetes/hypertension/liver diseases/CVD and the attitudes toward alcohol consumption was conducted in 2030 participants (6 participants were excluded due to lack of data regarding the diseases or attitudes toward alcohol consumption).

The individual health questionnaire contained following questions regarding alcohol consumption:

(1) Which best describes your history of alcohol use?
(2) At what age did you start drinking alcohol?
(3) What forms of alcohol have you regularly used?
(4) At least once a month, do you consume >5 alcoholic drinks/day?

In the first question the respondent could choose between “formerly used alcohol products”, “currently use alcohol products”, and “never used alcohol products”. In the third question, “regular use” was defined as at least once a month [25]. In the same question, the respondents were asked to assess the frequency of consumption (“daily”, “weekly”, “monthly”), the average number of drinks, duration of alcohol use (in years) of the following types and servings of alcohol: (a) spirits (rum, whiskey, gin, vodka, country liquor)—30 mL; (b) wine—125 mL; and beer—375 mL. In Poland, a country liquor is usually prepared by the method of maceration (with vodka or neutral spirits) of different ingredients (spices, herbs, fruit) with the addition of sugar. Typical polish country liquor contains 40–45% alcohol by volume, but some of them can be stronger. Due to the high content of alcohol, the country liquor was included in the category of spirits. The number of “drinks” that we refer to later was based on the aforementioned serving sizes of different types of alcohol. If the participants confirmed that they consume >5 alcoholic drinks per day at least once a month, they were asked two additional questions: (a) “How many times per month do you consume >5 alcoholic drinks in a day?” and (b) “What is the average number of drinks that you consume each time?”.

Following the methodology adopted in a study by Smyth et al. [27], participants who self-reported alcohol abstinence were defined as “never drinkers”. Participants who have ceased consumption of alcohol for at least 1 year prior to the interview were defined as “former drinkers”. The level of consumption of alcohol in current drinkers was defined as “low”, “moderate”, or “high”. “Low intake” was defined as drinking up to 7 drinks per week. “Moderate intake” was defined as drinking 7–14 drinks per week for women and 7–21 drinks for men. “High intake” was defined as drinking more than 14 drinks per week for women and more than 21 drinks per week for men. Heavy episodic drinking was defined as one episode of consumption of more than five drinks at least once per month. The number of drinks of each alcohol type was analyzed (beer, spirits, wine). The current drinker was included in the group for the type of drink according to which type they consumed most frequently. If participants declared the same frequency of consumption of more than one type of alcohol, they were defined as drinking “more than one type of alcohol” [27]. We also analyzed the association between attitudes toward alcohol consumption and the occurrence of diabetes, hypertension, CVD, and liver diseases. Diabetes was ascertained on the basis of (1) self-reported diabetes and/or (2) self-reported anti-diabetic medication and/or (3) fasting blood glucose measurement ≥ 126 mg/dL. Hypertension was ascertained on the basis of (1) self-reported hypertension and/or (2) self-reported anti-hypertensive medication and/or (3) an average of two blood pressure measurements ≥ 140/90 mmHg as previously described [28]. The category of “CVD” included coronary heart disease, stroke, heart infarction, heart failure, and other heart diseases. Liver diseases included hepatitis and jaundice. The occurrence of CVD and liver diseases were self-reported by the participants.

### 2.1. Statistical Analysis

Association between sociodemographic factors and consumption of alcohol was assessed with the use of logistic regression models after adjusting for age and/or gender. The strength of the association was measured by the odds ratio (OR) with 95% confidence intervals. The differences in the age of alcohol initiation between sexes and age groups were analyzed with the use of Mann–Whitney U and Kruskal–Wallis tests. Statistical analysis was done with the use of the program Statistica 13.1. We assumed a significance level of *p* < 0.05.

### 2.2. Ethics

The study was reviewed and accepted by the appropriate ethics committee and was therefore performed in accordance with the ethical standards laid down in an appropriate version of the 1964 Declaration of Helsinki (positive opinion of The Bioethics Committee of the Wrocław Medical University nr KB- 443/2006).

## 3. Results

### 3.1. Baseline Characteristics

At the baseline, a total of 2021 participants (747 men and 1274 women) were analyzed. The study group consisted of 17.5% of 30- to 44-year-olds, 66.4% of 45- to 64-year-olds, and 16.1% of participants aged 64 years and older. There were 59.3% of urban and 40.7% of rural participants. A total of 14.9% of participants had primary education, 16.0% had vocational education, 39.4% had secondary education, and 29.7% had higher education. At the baseline, 74.3% of participants were married/lived in a relationship, 18.5% of participants were separated/widowed/divorced, and 7.2% were never married. 

### 3.2. Alcohol Consumption

In the overall study population, 67.3% were current drinkers, 10.3% were former drinkers, and 22.4% declared that they had never drunk alcohol. Men were more likely than women to be current drinkers (77.2% vs. 61.5%, respectively) and former drinkers (11.6% vs. 9.5%, respectively). Almost one third of women (29.0%) and 11.1% of men declared that they had never drunk alcohol. The percentage of current drinkers decreased with increasing age (from 73.4% with age 30–44 years to 48.8% with age 64 and more). Current use of alcohol products was more prevalent in people living in an urban area (73.0%) and people with a higher level of education (78.0%). The chance of being a current drinker was nearly two times higher in people living in urban vs. rural areas (OR 1.88; CI 1.55–2.29). Moreover, the chance of being a current drinker was three times higher in participants with higher education than in people with primary education (OR 3.07; CI 2.25–4.20). We observed a lower proportion of current drinkers in divorced, separated, and widowed participants than in participants in a relationship (56.7% vs. 68.3% never married and 69.8% married) (Table 1).

Table 2 characterizes the current drinkers by the level of intake of alcohol products. The majority of participants (89.2%) declared a low level of alcohol intake, whereas only 1.8% declared a high level of intake. The chance of high level of intake of alcohol was four times higher in men than in women (OR 4.17; CI 1.64–10.6). The three-times greater chance of higher level of alcohol intake was also observed in participants with a higher level of education in comparison to participants with secondary education (OR 3.21; CI 1.15–8.99). Participants not living in a relationship also had a greater chance of a high level of alcohol consumption. Separated/divorced/widowed participants had a 3-fold higher chance and never married a 4.5-fold higher chance of higher level of alcohol intake than married participants (OR 2.95; CI 1.15–7.58 and OR 4.59; CI 1.58–13.3, respectively) (Table 2). 

Table 3 presents the prevalence of heavy episodic drinking in our cohort. Heavy episodic drinking occurred in 1 in 10 participants (9.9%). The chance of occurrence of heavy episodic drinking was two-fold higher in men than women (OR 2.41; CI 1.67–3.48). Heavy episodic drinking was also more prevalent in participants with vocational (OR 2.22; CI 1.01–4.91) and higher education (OR 2.29; CI 1.10–4.73) in comparison to participants with primary education. 

Table 4 presents the types of alcoholic beverages preferred by current drinkers in our cohort. The majority of participants (54.7%) declared most frequent consumption of low-alcohol drinks (wine, beer), a further 25.3% declared most frequent consumption of spirits, and the rest of the participants declared consumption of more than one type of alcohol. Spirits were chosen more frequently by women than men (25.8% vs. 24.7%, respectively), by participants aged 44 years or older (25.7% of participants aged 45–64 years; 39.2% of participants aged >64 years vs. 15.2% of participants aged 30–44 years). Spirits were also preferred by participants living in rural areas compared to in urban areas (30.5% vs. 22.4%, respectively) and participants with primary education (40.1%). Spirits were least frequently consumed by participants with a higher level of education (17.6%). 

**Figure 1:**
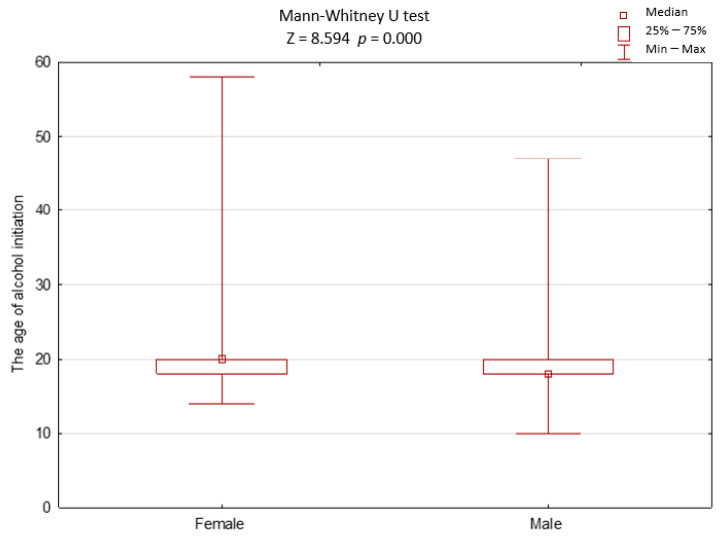
The age of alcohol initiation in women and men.

**Figure 2:**
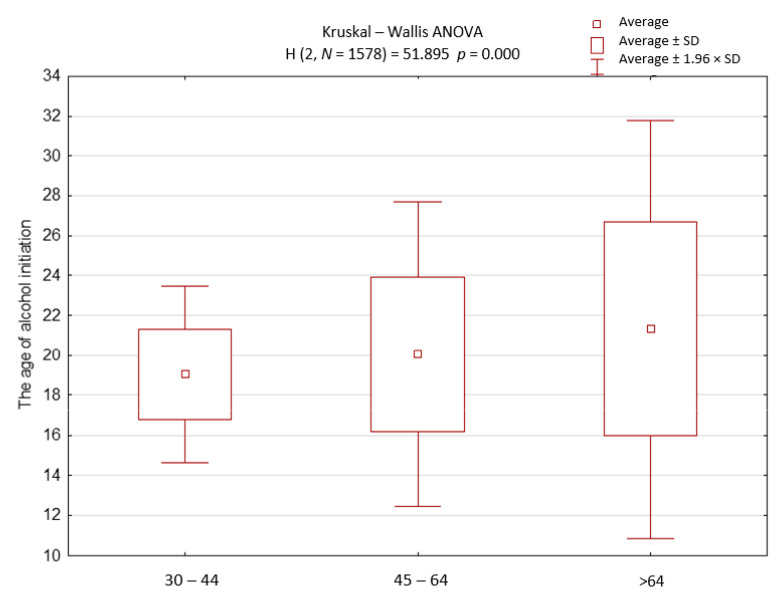
The age of the initiation of alcohol in different age groups.

The average age of initiation of alcohol consumption in our cohort was 20 ± 4 years (min 10, max 58). Sex and age were factors that statistically significantly differentiated the age of alcohol initiation. The initiation of alcohol occurred later in life in women than in men (Figure 1). The age of the initiation of alcohol decreased along with the decreasing age of participants (Figure 2).

**Figure 3:**
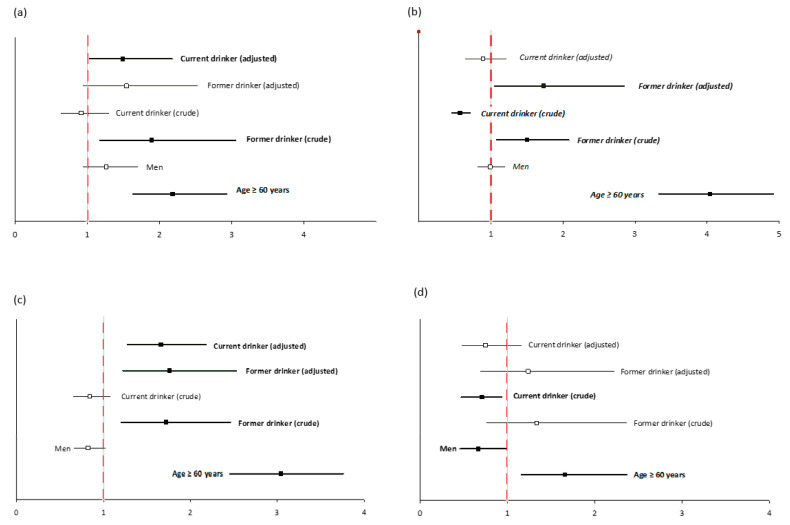
The odds ratio (with 95% CI) for occurrence of diabetes (**a**), hypertension (**b**), CVD (**c**), and hepatitis or jaundice (**d**) in groups of participants differentiated by attitudes toward alcohol consumption ((crude)—unadjusted OR; (adjusted)—attitudes toward alcohol consumption have been adjusted to age).

The odds ratio (with 95% CI) for occurrence of diabetes (a), hypertension (b), CVD (c), and hepatitis or jaundice (d) in groups of participants differentiated by attitudes toward alcohol consumption are presented in Figure 3. In the model, attitudes toward alcohol consumption have been adjusted to age. Current drinkers had almost 1.5-fold higher odds of diabetes than never drinkers (OR 1.49; CI 1.03–2.17). Former drinkers had higher odds for hypertension than never drinkers (1.73; CI 1.05–2.85). Both current and former drinkers had higher odds for CVD than never drinkers (OR 1.66, CI 1.27–2.18; OR 1.76, CI 1.22–2.53, respectively). We found no significantly higher odds for the occurrence of hepatitis/jaundice between current, former, and never drinkers. The odds of occurrence of diabetes, CVD, and hypertension were not significantly associated with sex. On the other hand, the odds of occurrence of hepatitis/jaundice were lower in men than women (OR 0.67; CI 0.46–0.99).

## 4. Discussion

This paper presents data about alcohol consumption patterns from one of the few cohort studies in Poland. Poland is one of the most interesting countries in which to investigate alcohol consumption patterns, due to rapid changes in attitudes toward alcohol over the years. It has been observed that since the 2000s, the level of alcohol consumption in Poland has steadily increased, exceeding the European average [12]. It is speculated that the increase in alcohol consumption might be contributing to a slower rate of health improvement in Poland [19,20]. The data regarding alcohol consumption coming from epidemiological studies are limited in Poland. In the National Multicenter Health Survey I (WOBASZ), conducted between 2003 and 2005, the most common type of alcohol consumed by men was beer, followed by vodka [29]. A total of 72% of men consumed 0.01–15.0 of pure ethanol per day [29]. The prevalence of current drinkers in our study was comparable to the percentage observed in the Polish–Norwegian Study (PONS) conducted between 2010 and 2011 (67.3 vs. 57.7%, respectively) [16]. Some discrepancies in a reported prevalence of attitudes towards alcohol consumption may be partially attributed to different definitions of “current alcohol drinkers” adopted in mentioned studies. According to the Public Opinion Research Center (CBOS), in 2019, a total of 56% of Poles drank alcoholic beverages at least occasionally and a further 8% declared drinking alcohol frequently [30]. In comparison to data obtained in 2010, there was a visible increase in occasional consumption of alcoholic beverages with a concomitant decrease in the percentage of both frequent drinkers and abstainers [30]. In the European region during the time period 2000–2016, there was a stable increase in the percentage of former drinkers (9.9% in 2000 vs. 16.6% in 2016) and a decrease in percentage of current drinkers (70.1% in 2000 vs. 59.9% in 2016) [1]. 

Our study revealed some sociodemographic tendencies in alcohol consumption patterns. We observed more current drinkers among men than women. Moreover, men had a tendency for a higher level of alcohol consumption and more frequent heavy episodic drinking than women. Similar observations were made worldwide [1] and in Europe [12,31]. Conforming to our results, in a cross-sectional study by Mierzecki et al. [15], women drank less frequently, declared significantly lower levels of alcohol consumption, and declared that they never consumed alcohol much more frequently than men. Similarly, in the PONS study [16], men reported more frequent drinking of alcoholic beverages than women. In an analysis of alcohol consumption patterns of global PURE study participants, people with high or moderate levels of alcohol consumption were predominantly older, male, and less educated [27]. According to the current status of research on alcohol consumption in Poland prepared by the CBOS, women drank much fewer alcoholic beverages than men and were abstainers more often, but there was a significant increase in the percentage of drinking women in Poland over the years [30]. In our study, the percentage of current drinkers decreased with increasing age of participants. A similar observation was made within the framework of the RARHA project, where the highest consumption of alcohol in Poland was noted among younger age groups and that the consumption decreased with the age of the participants [10]. Concomitantly, in our study, in the older age groups, the initiation of alcohol drinking occurred several years later than among the 30- to 44-year-olds and the proportion of abstainers and former drinkers was the highest in the oldest age group. The latter observation can be partially attributed to health-related problems occurring later in life which result in decreased alcohol consumption.

Another sociodemographic factor differentiating the patterns of alcohol consumption was the level of education. In our cohort, participants with higher education consumed more alcohol than those with a lower level of education. On the other hand, participants with a higher level of education chose predominantly low-alcohol beverages. In a study by Wojtyniak et al. [14], the association between level of education and consumption of alcohol was gender-specific. Urban residency and a higher level of education were associated with lower consumption of alcohol in men, but with higher consumption of alcohol in women [14]. A higher level of education was previously associated with a lower risk of heavy episodic drinking, a lower amount of alcohol consumed, and frequency of memory loss caused by drinking [13].

In our study, there were more current drinkers in the urban areas than in rural areas. The same observation was made in the PONS study [16]. Having said that, in our study, current drinkers living in urban areas declared more frequent consumption of low-alcohol beverages (wine, beer), whereas spirits were more prevalent in the rural areas. Contrary to our results, in a study by Mierzecki et al. [15], participants living in urban areas drank less often, consumed fewer alcoholic beverages, and had a lower frequency of heavy drinking than rural inhabitants. We observed no significant differences in the level of alcohol consumption and the prevalence of heavy episodic drinking between urban and rural inhabitants participating in our study.

It was estimated in 2018 that approximately one billion current drinkers worldwide were heavy episodic drinkers [1]. The prevalence of heavy episodic drinking among current drinkers in our cohort was similar to the prevalence observed in the global PURE study (9.9% vs. 13.1%, respectively) [27]. In contrast, the estimates made by the WHO for the European region were much higher. It was estimated that in Europe, heavy episodic drinking occurred in approximately 26.4% of population above 15 years of age (42.6% among drinkers) [1]. Europe was also the region with the highest prevalence of heavy episodic drinking [1]. The lower prevalence of heavy episodic drinking in our cohort can be partially explained by overrepresentation of participants with higher education in comparison to overall Polish population. Heavy episodic drinking is more common among people with a low socio-economic status.

There are some geographic differences when it comes to the type of preferred alcohol. Worldwide in 2018, the most commonly chosen type of alcohol was spirits (44.8%), but in the European region, the most consumed type of alcohol was beer (40.0%) [1,10]. In our cohort, beer was the most preferred type of alcohol, whereas spirits and wine were chosen equally frequently. According to data gathered by the State Agency for the Prevention of Alcohol-Related Problems (PARPA), beer was the most common type of alcohol beverage chosen by Poles in 2019 (54.6%), followed by spirits (37.8%) and wine (7.6%) [18]. Similarly, beer was the dominant type of alcohol consumed by Poles in the RARHA project [10]. In contrast, in the PONS study, the most frequently chosen type of alcohol was vodka and other spirits (75.4%), followed by wine (61.5%) and beer (54.0%) [16]. In a self-reported preference of type of alcoholic beverages prepared by CBOS in 2019, Poles chose predominantly beer (39%), then wine (25%) and spirits (16%) [30]. Conforming to our results, men preferred mostly beer (56%), whereas women preferred wine (45%) [30]. We noted differences in the preferred types of alcohol between the age groups. Participants in the youngest age group (30- to 44-year-olds) preferred beer, whereas spirits were the least popular type of alcohol in this age group. In contrast, the oldest participants (>64 years of age) preferred spirits over other types of alcohol. Similar observations can be found in the report of the RARHA project, where beer was the most common type of alcohol among younger participants [10]. The shift in the preferences is worth noting, but it is also expected. Several decades ago in Poland, spirits were the most accessible type of alcohol. The increasing consumption of beer and other low-alcohol beverages over spirits has been observed in Poland since the 1990s [32]. According to the report prepared by the Nielsen Company on the condition of the alcohol market in Poland, there was a similar increase in the sales of beer and spirits over the last years, but beer and low-alcohol beverages were still a leading category on the market [33]. It was also observed that there was an increase in interest in high quality alcoholic beverages, like cognac, brandy, or whiskey [30,33].

In our study, current drinkers had higher odds for diabetes and CVD, whereas former drinkers had higher odds for CVD and hypertension than never drinkers. This observation might suggest that giving up alcohol consumption might be related to deteriorating health. Former drinkers were considered less healthy than never drinkers and have a higher mortality rate [34]. The possible bias of considering former drinkers as abstainers and the decline in alcohol consumption along with progressing age and deterioration of health has been previously reported [35,36,37,38,39,40]. We are reporting the attitudes toward alcohol consumption and occurrence of diseases at the same point in time, so we are unable to determine causality between those events. In a meta-analysis performed by Baliunas et al. [41], a U-shape association between alcohol consumption and the risk of type 2 diabetes was observed. A moderate consumption of alcohol has been associated with a lower risk of type 2 diabetes (the most protective effect observed in case of consumption of 22 g of alcohol per day in men and 24 g of alcohol per day in women) [41]. It has been proposed however, that the favorable effect of alcohol on the diabetes risk in this meta-analysis could have resulted from some bias, e.g., including in the reference group both never drinkers and less healthy former drinkers [42]. In a more u p-to-date meta-analysis conducted by Knott et al., no reduction in diabetes risk was observed in men, regardless of the level of alcohol consumption [42]. On the other hand, a reduction in the risk of type 2 diabetes was observed in women (peak reduction at the level of consumption of 31–37 g of alcohol per day) [42]. Having said that, it was observed that among the patients with diagnosed diabetes, limiting alcohol consumption by at least 2 units/week decreased a 10-year risk of CVD (HR: 0.56, 95% CI 0.36, 0.87) [43]. Alcohol consumption is also a well-known risk factor for hypertension [44,45,46]. Heavy drinking has been associated with an increased risk of hemorrhagic stroke [47,48]. In the analysis of the modifiable risk factors of CVD and mortality in the global PURE study, high alcohol drinkers showed a moderate increase in CVD incidence, but a high increase in overall mortality [49]. Low-alcohol drinkers also had a lower risk of CVD incidence than abstainers [49]. In a study by Bell et al. [50], heavy drinkers and former drinkers had a higher risk of coronary death, heart failure, cardiac arrest, transient ischemic attack, ischemic stroke, intracerebral hemorrhage, and peripheral arterial disease. In the same study, abstaining from alcohol was associated with an increased risk of unstable angina, myocardial infarction, heart failure, stroke, and peripheral arterial disease in comparison to moderate drinking [50]. In a current analysis of 599,912 participants from 83 prospective studies, the lowest risk of mortality in current drinkers was observed with the consumption of 100 g of alcohol per week [51]. In the same study, when considering CVD subtypes, increased alcohol consumption was linearly associated with a higher risk of stroke and coronary artery disease [51]. For CVDs other than myocardial infarction, there was no threshold below which lower alcohol consumption ceased to be associated with a lower risk [51]. Taken together, although the impact of alcohol consumption on CVD is controversial, the position of the European Society of Cardiology is that the current limits of alcohol consumption should be lowered because the harm outweighs the possible benefits of moderate drinking [52].

There are some limitations of our study to consider. Firstly, this is a cohort study and although the population sample is quite large, the results should be treated with caution as they cannot be exactly extrapolated to the whole Polish population. The study group was not randomized, which can introduce a possible bias. Our cohort is characterized by an overrepresentation of women, elderly people, and better educated people in comparison to the Polish population overall. It is important to note that self-reported alcohol consumption can be underestimated and that there is a risk of bias. On the other hand, as stated previously in the literature, self-reported alcohol consumption, although not without some issues, is a reliable and acceptable method, especially in large-scale studies [53]. The risk of bias in our study is lowered by the fact that the questionnaires were conducted by a trained researcher. We present only the data collected at the baseline. On the other hand, there are currently only a few large cohort studies that have been conducted in Poland. Directions for the future research include a longitudinal prospective analysis of alcohol consumption patterns in the PURE Poland cohort study.

## 5. Conclusions

In our cohort study, we observed several socio-demographic factors differentiating the patterns of alcohol consumption. Participants living in urban areas and with a higher level of education drank more alcohol, but in contrast to rural inhabitants and participants with a lower level of education, they chose mostly low-alcohol beverages. Current drinkers had higher odds for diabetes and CVD, whereas former drinkers had higher odds for CVD and hypertension than never drinkers.

## Figures and Tables

**Table 1:** Sociodemographic characteristics of attitudes toward alcohol drinking in the PURE Poland cohort study.

Characteristics	Currently Use Alcohol Products % (*n*)	Formerly Used Alcohol Products % (*n*)	Never Used Alcohol Products % (*n*)	*p* ***	OR (95% CI)
Total	67.3 (1360)	10.3 (208)	22.4 (453)		
Sex					
Men	77.2 (577)	11.6 (87)	11.1 (83)	<0.001	2.10 (1.71 to 2.58) ^a^
Women	61.5 (783)	9.5 (121)	29.0 (370)		Ref.
Age					
30–44	73.4 (260)	8.2 (29)	18.4 (65)	<0.001	Ref.
45–64	70.2 (941)	8.6 (115)	21.3 (285)		0.45 (0.35 to 0.57) ^b^
>64	48.8 (159)	19.6 (64)	31.6 (103)		0.39 (0.27 to 0.55) ^b^
Place of residence					
Urban	73.0 (874)	7.2 (86)	19.8 (238)	<0.001	1.88 (1.55 to 2.29) ^c^
Rural	59.1 (486)	14.8 (122)	26.1 (215)		Ref.
Level of education *					
Primary	52.2 (157)	20.6 (62)	27.2 (82)	<0.001	Ref.
Vocational	65.2 (210)	12.1 (39)	22.7 (73)		1.57 (1.12 to 2.22) ^c^
Secondary	66.1 (525)	9.6 (76)	24.3 (193)		1.83 (1.38 to 2.41) ^c^
Higher	78.0 (467)	5.0 (30)	17.0 (102)		3.07 (2.25 to 4.20) ^c^
Marital status **					
Married/living together	69.8 (1048)	9.3 (139)	20.9 (314)	<0.001	Ref.
Separated/divorced/widowed	56.7 (212)	14.4 (54)	28.9 (108)		0.68 (0.53 to 0.88) ^c^
Never married	68.3 (99)	10.3 (15)	21.4 (31)		0.93 (0.64 to 1.35) ^c^

* 5 participants were excluded due to a lack of information about their level of education; ** 1 participant was excluded due to a lack of information about their marital status; *** Chi-square test; OR—being a current drinker, ^a^—OR_adj._—odds ratio adjusted for age, ^b^—OR_adj._—odds ratio adjusted for sex, ^c^—OR_adj._—odds ratio adjusted for sex and age.

**Table 2:** Baseline characteristics in current drinkers by level of intake alcohol.

Characteristics	Number of Drinks Me (IQR)	*p* ***	Alcohol Consumption Intensity % (*n*)			*p* ****	OR (95% CI)
			High	Moderate	Low		
Total	0.8		1.8	9.0	89.2		
	(0.2–2.0)		(24)	(122)	(1214)		
Sex							
Men	1.5	0.001	3.1	18.0	78.9	<0.001	4.17
	(0.5–4.8)		(18)	(104)	(455)		(1.64–10.6) ^a^
Women	0.5		0.8	2.3	96.9		Ref.
	(0.2–1.0)		(6)	(18)	(759)		
Age							
30–44	1.3	<0.001	2.3	10.0	87.7	0.888	1.00 (ref.)
	(0.5–3.5)		(6)	(26)	(228)		
45–64	1.0		1.6	8.8	89.6		0.69
	(0.5–2.3)		(15)	(83)	(843)		(0.26–1.78) ^b^
>64	0.8		1.9	8.2	89.9		0.81
	(0.3–2.0)		(3)	(13)	(143)		(0.20–3.30) ^b^
Place of residence							
Urban	1.0	>0.05	1.8	8.6	89.6	0.230	0.90
	(0.5–2.5)		(16)	(75)	(783)		(0.38–2.11) ^c^
Rural	1.0		1.6	9.7	88.7		Ref.
	(0.5–2.3)		(8)	(47)	(431)		
Level of education *							
Primary	0.8	<0.001	0.6	8.3	91.1	0.084	0.67
	(0.5–2.0)		(1)	(13)	(143)		(0.08–5.75) ^c^
Vocational	1.0		1.9	7.1	91.0		2.02
	(0.5–3.0)		(4)	(15)	(191)		(0.54–7.60) ^c^
Secondary	1.0		1.0	8.2	90.9		Ref.
	(0.5–2.0)		(5)	(43)	(477)		
Higher	1.3		3.0	10.9	86.1		3.21
	(0.5–3.0)		(14)	(51)	(402)		(1.15–8.99) ^c^
Marital status **							
Married/living together	1.0	0.014	1.0	9.8	89.2	0.002	Ref.
	(0.5–2.5)		(12)	(103)	(933)		
Separated/divorced/widowed	0.8		3.3	4.7	92.0		2.95
	(0.5–1.5)		(7)	(10)	(195)		(1.15–7.58) ^c^
Never married	1.0		5.1	9.1	85.9		4.59
	(0.5–3.0)		(5)	(9)	(85)		(1.58–13.3) ^c^

* 5 participants were excluded due to a lack of information about their level of education; ** 1 participant was excluded due to a lack of information about their marital status; *** ANOVA—analysis of variance; **** Chi-square test; OR—higher alcohol consumption, ^a^—OR_adj._—odds ratio adjusted for age, ^b^—OR_adj._—odds ratio adjusted for sex, ^c^—OR_adj._—odds ratio adjusted for sex and age.

**Table 3:** The baseline prevalence of heavy episodic drinking among participants who declared current use of alcohol products.

Characteristics	Prevalence		*p* ***	OR
	Yes	No		
**Total**	9.9	90.1		
	(134)	(1226)		
Sex				
Men	14.4	85.6	<0.001	2.41 (1.67–3.48) ^a^
	(83)	(494)		
Women	6.5	93.5		Ref.
	(51)	(732)		
Age				
30–44	11.5	88.5	0.409	Ref.
	(30)	(230)		
45–64	9.8	90.2		0.83 (0.54–1.29) ^b^
	(92)	(849)		
>64	7.5	92.5		0.63 (0.31–1.26) ^b^
	(12)	(147)		
Place of residence				
Urban	10.2	89.8	0.584	1.11 (0.76–1.62) ^c^
	(89)	(785)		
Rural	9.3	90.7		Ref.
	(45)	(441)		
Level of education *				
Primary	5.7	94.3	0.036	1.00 (ref.)
	(9)	(148)		
Vocational	11.9	88.1		2.22 (1.01–4.91) ^c^
	(25)	(185)		
Secondary	8.2	91.8		1.47 (0.70–3.08) ^c^
	(43)	(482)		
Higher	12.2	87.8		2.29 (1.10–4.73) ^c^
	(57)	(410)		
Marital status **				
Married/living together	10.3	89.7	0.596	1.00 (ref.)
	(108)	(940)		
Separated/ divorced/widowed	8.5	91.5		0.81 (0.48–1.36) ^c^
	(18)	(194)		
Never married	8.1	91.9		0.77 (0.36–1.62) ^c^
	(8)	(91)		

* 5 participants were excluded due to a lack of information about their level of education; ** 1 participant was excluded due to a lack of information about their marital status; *** Chi-square test, OR—heavy episodic drinking, ^a^—OR_adj._—odds ratio adjusted for age, ^b^—OR_adj._—odds ratio adjusted for sex, ^c^—OR_adj._—odds ratio adjusted for sex and age.

**Table 4:** The baseline characteristics of the types of alcoholic beverages most frequently consumed by participants who declared current use of alcohol products.

Characteristics	Spirits % (*n*)	Wine % (*n*)	Beer % (*n*)	More Than One Type of Alcohol % (*n*)	*p* ***
**Total**	25.3	25.2	29.5	20.0	
	(343)	(342)	(399)	(271)	
**Sex**					
**Men**	24.7	12.9	49.4	13.0	<0.001
	(142)	(74)	(284)	(75)	
**Women**	25.8	34.4	14.7	25.1	
	(201)	(268)	(115)	(196)	
**Age**					
**30–44**	15.2	28.4	41.2	15.2	<0.001
	(39)	(73)	(106)	(39)	
**45–64**	25.7	24.7	28.3	21.3	
	(242)	(232)	(266)	(200)	
**>64**	39.2	23.4	17.1	20.3	
	(62)	(37)	(27)	(32)	
**Place of residence**					
**Urban**	22.4	28.6	28.3	20.7	<0.001
	(195)	(249)	(246)	(180)	
**Rural**	30.5	19.2	31.5	18.8	
	(148)	(93)	(153)	(91)	
Level of education *					
**Primary**	40.1	12.7	28.7	18.5	<0.001
	(63)	(20)	(45)	(29)	
**Vocational**	27.6	18.1	38.6	15.7	
	(58)	(38)	(81)	(33)	
**Secondary**	26.8	24.5	27.6	21.1	
	(140)	(128)	(144)	(110)	
**Higher**	17.6	33.5	27.6	21.3	
	(82)	(156)	(128)	(99)	
Marital status **					
**Married/living together**	24.5	24.5	31.7	19.3	0.03
	(256)	(256)	(331)	(201)	
**Separated/divorced/widowed**	28.0	28.4	19.4	24.2	
	(59)	(60)	(41)	(51)	
**Never married**	28.3	26.3	27.3	18.2	
	(28)	(26)	(27)	(18)	

* 5 participants were excluded due to a lack of information about their level of education; ** 1 participant was excluded due to a lack of information about their marital status; *** Chi-square test.

## Data Availability

Data available on request due to restrictions, e.g., privacy or ethical.
